# A MAC Protocol for Medical Monitoring Applications of Wireless Body Area Networks

**DOI:** 10.3390/s150612906

**Published:** 2015-06-03

**Authors:** Minglei Shu, Dongfeng Yuan, Chongqing Zhang, Yinglong Wang, Changfang Chen

**Affiliations:** 1 School of Information Science and Engineering, Shandong University, Jinan 250100, China; E-Mails: shuml@sdas.org (M.S.); dfyuan@sdu.edu.cn (D.Y.); 2 Shandong Provincial Key Laboratory of Computer Networks, Shandong Computer Science Center (National Supercomputer Center in Jinan), Jinan 250101, China; E-Mails: wangyl@sdas.org (Y.W.); chenchangfang012@163.com (C.C.); 3 College of Information Science and Engineering, Shandong University of Science and Technology, Qingdao 266590, China

**Keywords:** MAC protocol, wireless body area networks (WBANs), medical monitoring, I-MAC

## Abstract

Targeting the medical monitoring applications of wireless body area networks (WBANs), a hybrid medium access control protocol using an interrupt mechanism (I-MAC) is proposed to improve the energy and time slot utilization efficiency and to meet the data delivery delay requirement at the same time. Unlike existing hybrid MAC protocols, a superframe structure with a longer length is adopted to avoid unnecessary beacons. The time slots are mostly allocated to nodes with periodic data sources. Short interruption slots are inserted into the superframe to convey the urgent data and to guarantee the real-time requirements of these data. During these interruption slots, the coordinator can break the running superframe and start a new superframe. A contention access period (CAP) is only activated when there are more data that need to be delivered. Experimental results show the effectiveness of the proposed MAC protocol in WBANs with low urgent traffic.

## 1. Introduction

The advances in the miniaturization of electronic devices, intelligent monitoring sensors, battery and wireless communication technologies have boosted the development of wireless body area networks (WBANs). WBANs are promising for a wide range of applications, among which healthcare is an important application [1]. By adopting small-sized, wearable or implanted sensors or actuators with a wireless communication ability, WBAN technology can realize a new ambulatory monitoring and treatment model for patients. Compared to the traditional monitoring and treatment approach, the new monitoring and treatment model can greatly free the activities of patients and does not bring much interference to the daily routines of the patients [2].

Medical monitoring is one typical kind of applications of WBANs. WBANs can realize continuous or prolonged monitoring of many chronic or non-chronic diseases, e.g., cardiovascular disease (CVD), diabetes, Alzheimer's disease, Parkinson's disease, *etc*. [3]. Generally speaking, there is only one coordinator in such a WBAN, whereas the number of nodes may range from zero to several dozens. A WBAN generally adopts a star topology in which the coordinator acts as a master and the peripheral nodes act as the slaves. The coordinator also serves as the data sink, coordinating the communications in the WBAN. The nodes measure the temperature, blood pressure, electrocardiogram (ECG), electroencephalogram (EEG), *etc*., and then deliver the collected data to the coordinator [4]. The coordinator then forwards the received data to a monitoring station or other data management infrastructure [5].

In a WBAN medical monitoring application, different sensor nodes connected inside or on the body are frequently requested to collect body data periodically and then send the data to the coordinator. These periodic data may have great variations in terms of sensing rate and delivery latency. On the one hand, these variations can be caused by the monitoring of different physiological sources, such as ECG, oxygen, body temperature, blood pressure, multimedia data, *etc*., and on the other hand, each sensor itself can have significant variations according to the demands of the applications, the state of health of the patient, *etc*. A summary of the data rate and delivery latency demands of different types of data is given in Table 1 [1,3].

Besides the periodic data, there exist many types of data, e.g., physiological emergencies data [6,7], network command frames, *etc*. Different from periodic data, data are generated and sent only when certain events happen, e.g., a sensor detects the occurrence of a stroke or a sensor applies for several guaranteed time slots (GTS), *etc*. Because these data generally are urgent, we call these data urgent data in this paper. Actually, many types of traditional periodic data can be transformed to urgent data. For example, by changing the periodic monitoring of body temperature to report it when it is out of normal bounds, the periodic collecting of temperature data is changed to urgent reporting of temperature data. With the potential of immensely reducing the amount of data or traffic, this method can be an effective approach to save energy and extend the node life.

Periodic data and urgent data have different data rates and quality of service (QoS) demands. From Table 1, many periodic applications generate high traffic, which means a large amount of communication resources is needed to accommodate this traffic. The real-time requirements of periodic data are also high. Yet, the reliability requirements of periodic data are generally not high, especially for multimedia data. Compared with periodic data, the data rate of urgent data is generally very low. Yet, the real-time and reliability requirements of urgent data may be fairly high [6,7]. For example, physiological emergency events should be reported to the doctors and nurses reliably and as quickly as possible, and network commands should be transmitted quickly and reliably so that the network can work effectively.

Two MAC mechanisms, carrier sense multiple access with collision avoidance (CSMA/CA) and time-division multiple access (TDMA), are mainly used to accommodate the variations of the periodic data and urgent traffic in WBANs [8]. CSMA/CA is a contention-based channel access mechanism, whereas TDMA is a contention-free mechanism. Using CSMA/CA, a node needs to compete with other nodes during the contention access period (CAP) to get access to the channel. While using TDMA, a node can transmit its packets in a collision-free manner using time slots allocated to it. Both mechanisms have different advantages and disadvantages. As reported in [8], a comparison between TDMA and CSMA/CA protocols is given in Table 2.

From Table 2, TDMA outperforms CSMA/CA on power consumption, bandwidth utilization and preferred traffic level [8]. Yet, it has some shortcomings resulting from its disability for adapting to network dynamics and its dependency on time synchronization. Researchers have come to a common view that the CSMA/CA mechanism is more suitable for networks with low, urgent traffic and high network dynamics, while TDMA is more suitable for networks with high, periodic traffic and low network dynamics [8]. With a simple star structure composed of a coordinator and sensor nodes, a WBAN is generally not of high dynamics, and the simple structure simplifies time synchronization. Periodic data dominate the whole traffic in general. Therefore, TDMA is propitious to transmit the periodic data. However, this does not mean the network dynamics and urgent traffic could be ignored. CSMA/CA still can find its role to accommodate these dynamics and traffic.

The network dynamics of a WBAN can be caused by joins or departures of sensor nodes, breakdowns of nodes or the coordinator, temporary break offs of communication resulting from body motions or other reasons. The urgent data in a WBAN include physiological emergencies data, user commands, network command frames, *etc*. Many hybrid MAC protocols that combine CSMA/CA and TDMA schemes have been designed to meet the above demands, e.g., 802.15.4 [9], 802.15.6 [10], *etc*. Using such a scheme, TDMA is adopted to reduce collisions and conserve energy for channel access. CSMA/CA is used to convey emergencies’ data and network commands. Such a network generally operates in a beacon-enabled mode in which the time synchronization is performed by the help of a beacon frame.

A hybrid MAC protocol [11], e.g., 802.15.4, can be adopted to meet the demands of a WBAN. A superframe of such a MAC protocol generally contains two kinds of periods, contention access period (CAP) and contention-free period (CFP). Each period is composed of one or many basic time slots. During the CAP period, a node competes with other nodes to transmit emergencies data and network commands, *etc*. The CFP period is further divided into multiple periods, which are assigned to different nodes. Upon being assigned one or several CFP slots, a node can use these slots to transmit its data, and it can turn off its antenna to save energy during the CAP period and other CFP slots. By combining CSMA/CA and TDMA together, a hybrid MAC protocol can achieve a higher medium access performance. However, there are still several shortcomings concerning such a hybrid MAC protocol, e.g., 802.15.4. Using a hybrid MAC protocol, the urgent data are conveyed during the CAP period. How much time should be allocated as the CAP period is a problem. More time means the waste of communication resources, and less time may incur a long delay of frames and even the loss of frames. Urgent data generally have high real-time requirements, which implies that the length of a superframe should be short enough to guarantee the real-time demands. Thus, beacon frames need to be broadcast frequently. This means too many unnecessary beacon frames are transmitted and received, and this results in unnecessary energy waste and communication resource waste.

In this paper, a hybrid MAC protocol using interrupt mechanism (I-MAC) is proposed to solve the above-mentioned problems. Unlike existing hybrid MAC protocols, I-MAC uses a superframe structure with a longer length to avoid unnecessary beacons. The time slots are mostly allocated to nodes with periodic data sources. To convey urgent data and guarantee the real-time requirements of these data, short interrupt slots are inserted between the time slots assigned to nodes with periodic data sources. During these slots, the coordinator can break the running superframe and start a new superframe. A CAP period is only activated and used to convey urgent data when there are more data that need to be delivered. In a WBAN with low urgent traffic, the proposed MAC protocol can improve the use ratio of time slots, reduce the energy consumption and satisfy the real-time requirements at the same time. The experimental results show the effectiveness of the proposed MAC protocol.

The next section introduces the related works. Section 3 sets out the network model and assumptions and introduces the superframe structure and operations of the proposed MAC protocol. Section 4 presents a numerical analysis of the energy efficiency, time slot utilization efficiency and data delivery delay for 802.15.4 and the proposed protocol. The performances of 802.15.4 and the proposed protocol are evaluated in Section 5. Finally, conclusions are drawn in Section 6.

## 2. Related Works

MAC protocols play an indispensable role in ensuring that WSNs and WBANs function properly. Many MAC protocols have been proposed to extend the network life of WSNs, e.g., S-MAC [12], T-MAC [13], WiseMAC [14], B-MAC [15], TRAMA [16], and so on. However, all of these protocols show inadequate network throughput and delay performance for varying traffic, which is common in WBANs.

The MAC layer in WBANs has been a hot topic that has drawn a lot of attention in the last few years. Aiming at the special characteristics of WBANs, much work has been done to design new MAC protocols [17–20] or to revise IEEE 802.15.4 [21–23] to meet different demands. In [24], a cloud-assisted RLNC-based MAC protocol was proposed to speed up the information flow between patient-worn sensors and the medical data center. In [25,26], a MAC protocol and a QoS control scheme are specially designed for energy-harvesting WBANs. However, these protocols are not optimized for transmitting the emergency data or the other urgent data. As the standard of WBANs, IEEE 802.15.6 is a versatile communication suite that can adapt to many kinds of application. Yet, it only defines the operation elements that enable the interoperations of the WBAN devices, and the users are supposed to customize the 802.15.6 according to the demands of the practical application. In this light, the MAC scheme of 802.15.6 is not a complete MAC protocol [27].

Several MAC protocols [6,7,28,29] have been proposed to handle an emergency event in WBANs. However, these protocols did not take the other urgent data into consideration. The existing work most similar to our work is [30], in which the authors proposed an adaptive beaconing MAC protocol (AB-MAC) to provide healthcare services. Standby slots and access slots are adopted by AB-MAC to identify the event-driven data, and then AB-MAC adaptively broadcasts a new beacon frame and reschedules the time slots. However, AB-MAC has several drawbacks. First, the access slot used by AB-MAC is too long and is not fully utilized, thus AB-MAC can only be employed in a WBAN with several nodes. Second, AB-MAC did not consider the priority of events, so an event with low priority may preempt an event with high priority. Third, AB-MAC did not take the length of the data into consideration; thus, any event will lead to an access slot and an adaptive beacon, which will impair the energy efficiency and communication efficiency.

## 3. MAC Protocol Design

### 3.1. Network Model and Assumptions

The target applications of the proposed MAC protocol are medical monitoring applications of wireless body area networks. Such a network adopts a star topology with *N* heterogeneous nodes and a single coordinator. The nodes are powered with batteries that are not replaced or recharged conveniently. The coordinator is assumed to be a device equipped with a rechargeable battery, which can be recharged conveniently [31]. Thus, the coordinator is not subject to the same energy constraint as the nodes. Each node is within the carrier sensing range of the other nodes. Time is divided into superframes, each of which has a beacon frame and a period for communication. A node only monitors one physiological or physical phenomenon or object. This conforms to the present status of the WBAN industry.

It is assumed that there are three kinds of monitoring schemes in a WBAN. The first scheme is monitoring the target and reporting the data for a certain period and with a certain frequency, e.g., monitoring EEG and reporting the data for one hour with an interval of 1 s. This scheme generally generates high periodic traffic, which needs plenty of communication resource. The second scheme still needs to monitor the target for a certain period and with a certain frequency, but the data are reported to the coordinator conditionally, e.g., when certain emergencies happen or the data are out of predefined bounds, *etc*. The third scheme is monitoring the object once and reporting the data to the coordinator. The traffic generated by the second and third schemes is sporadic and low. The packet rate may be one-tenth of a packet per second (pps) or even lower, and the payload of a packet may be several bytes to several kilobytes. This depends on the data rate of the sensor nodes and the actual condition of the patient being monitored. The real-time and reliability requirements of different urgent data may be different. For example, the emergency data have extremely high real-time and reliability requirements, while the same requirements of a body temperature data can be quite low. Beside the traffic brought by the above three monitoring schemes, user commands and network commands also lead to a small amount of traffic. The real-time and reliability requirements of these commands are also fairly high.

Based on the above network model and assumptions, the purpose of this paper is to design a MAC protocol for the WBAN medical monitoring applications mentioned above. The proposed protocol can meet the above-mentioned real-time and reliability requirements and possesses high efficiencies for making use of energy and communication resources at the same time. The goal of the protocol is to have the following characteristics.

1. High efficiency for utilizing time slots: in a beacon-enabled WBAN, time slots are the main communication resource. From the above discussion, periodic data give rise to far more traffic than urgent data. On the basis of reserving enough time slots for the urgent data, the proposed MAC protocol assigns time slots to nodes with mass periodic data as much as possible to improve the efficiency of utilizing the time slots. An additional CAP period is only activated and used to convey urgent data when there are more data that need to be delivered. Big urgent data are also conveyed using GTS allocated according to the size of the data.
2. Real-time communication: the real-time transmission of periodic data is provided using the guaranteed time slots (GTS). Interrupt time slots are inserted between the GTS to convey urgent data. The coordinator can adjust the interval between interrupt time slots according to the frequency of urgent data. When big urgent data with high priority are generated and reported, the coordinator can break the currently running superframe and start a new superframe to convey the data first. These measures can guarantee the real-time communication of the periodic and urgent data.
3. High energy efficiency: to transmit the urgent data with a low delay, existing MAC protocols employ a short superframe length to guarantee the real-time demands. This means unnecessary transmitting and receiving of beacon frames, which results in unnecessary energy waste and communication resource waste. By adopting a long superframe length and interrupt time slots, energy spent on unnecessary transmitting and receiving of beacon frames can be saved while the real-time demands still can be guaranteed.
4. Reliable communication: some urgent data require high reliability. The proposed MAC protocol uses an acknowledgment and retransmission mechanism to meet the reliability requirements. An interrupt time slot is divided into two sections, one of which is used to transmit urgent data or network commands and the other to acknowledge the transmission.

### 3.2. The Superframe Structure

**Figure 1:**
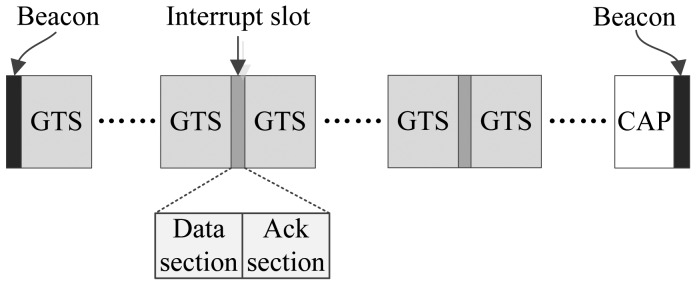
Structure of the I-MAC superframe.

The superframe structure of the proposed MAC protocol is shown in Figure 1. Like 802.15.4, the beginning of the superframe is a beacon frame, which contains information, such as the timestamp, beacon interval, time slot assignments, *etc*. Just as mentioned above, one purpose of the proposed protocol is to assign the time slots to nodes that are generating busy traffic as much as possible. To meet this end, most of the whole superframe is mapped out to be GTS that can be assigned to nodes that run periodic monitoring applications of high traffic or to nodes that have big urgent data to transmit.

Following the beacon frame, there may exist several optional GTS that are dedicated to nodes with big urgent data. The existence of these GTS depends on the foregoing superframe. If there were big urgent data generated during the forgoing superframe and the owner node of the data has sent a GTS request, then the optional GTS will be allocated and assigned to the node for conveying the big urgent data. Other GTS are distributed to nodes that generate periodic data. These GTS form a loop structure according to the data rates of the nodes.

Between some GTS, interrupt slots are inserted to deliver the urgent data. These slots interrupt the running superframe and provide the coordinator and nodes with opportunities to transmit their data or commands. In this sense, the mechanism of an interrupt slot is similar to some interrupt mechanisms used in computer. Thus, we call these time slots interrupt slots, and the proposed MAC protocol is called I-MAC correspondingly. The interval between two neighboring interrupt slots is fixed in a superframe. Yet, the interrupt intervals of different superframes can be different. The interrupt intervals are calculated based on the production of the urgent data. There is more information about this in the following content.

At the end of a superframe is an optional CAP period. Whether this CAP period exists also depends on if there are collisions happening or not. If there is more than one urgent data frame colliding in one interrupt slot, such a CAP period will be activated to retransmit these urgent data frames. It can be seen that the superframe structure of the proposed protocol is more complex than IEEE 802.15.4, hence more bytes are needed to implement and describe such a superframe structure. As a result, the beacon length of I-MAC is longer than 802.15.4.

From Figure 1, an interrupt slot can be seen that is composed of two sections, a data section and an acksection. The data section is designed to be used by a node to send its one urgent datum, e.g., an emergency datum or a GTS request, to the coordinator, or by the coordinator to send the user commands or network commands to the nodes. The ack section is supposed to be used to acknowledge the data or commands. In addition to this, the ack section can also be used by the coordinator to send special “BREAK”, “CAP” and “SYNC” commands to all of the nodes. On receiving a “BREAK” command frame, all nodes break the current superframe, wait for the end of the ack section and begin to receive a new beacon frame. This means the start of a new superframe.

On receiving a “CAP” command frame, all nodes wait for the end of the ack section and then enter a CAP period, in which all of the nodes compete for transmitting their data. This mechanism is similar to the adaptive CAP mechanism in 802.15.6 [10]. The length of the CAP period is contained in the “CAP” command frame. A “SYNC” command frame is used to transmit time synchronization information to the nodes. On receiving a “SYNC” command frame, each of the nodes retrieve the frame field and uses the contained synchronization data to synchronize its clock with the coordinator.

There are two types of interrupt time slots: one is used for the coordinator to transmit data packets or commands to nodes, and the other one is used for nodes to transmit data packets or commands to the coordinator. We call the first type of interrupt slots “download” interrupt slots and the second type of interrupt slots “upload” interrupt slots. The difference between two types of interrupt slots lies in the sender and receiver during different sections. For a download interrupt slot, during the data section, the coordinator acts as the sender, and all nodes act as receivers. From this, it can be deduced that the download interrupt slot can be used by the coordinator for broadcasting messages to nodes. During the ack section of a download interrupt slot, the coordinator acts as the receiver, and some node acts as the sender. Only unicasts are acknowledged, and broadcasts are not acknowledged. A download interrupt slot can also be used by the coordinator to send “BREAK”, “CAP” and “SYNC” commands to all of the nodes.

**Figure 2:**
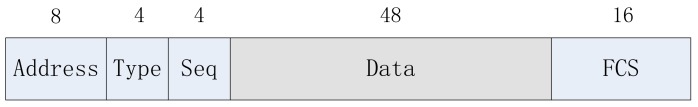
Structure of the interrupt data frame. Seq, sequence; FCS, frame check sequence.

The structure of the frame transmitted in the data section of an interrupt slot is illustrated in Figure 2. Such a frame is called an interrupt data frame. As the figure shows, the total length of the frame is 80 bits, *i.e.*, 10 bytes. The address field indicates the sender address if the frame is from a node to the coordinator. On the contrary, this field indicates the receiver address if the frame is from the coordinator to one node. It should be noted that if the frame is a broadcast frame, then the address should be set to be the broadcast address, which is 255 in this paper. The type field denotes the data type carried by the frame. The Seq field contains a sequence number that can be used for acknowledgment. The data field is six bytes long. This length is enough for the network commands and urgent data, like body temperature, blood pressure, blood glucose, emergencies event data, *etc*. Certainly, there are urgent data that cannot be encapsulated into one interrupt data frame. In this paper, we call the urgent data that can be carried by one interrupt data frame small data and the urgent data that can be carried by one interrupt data frame big data. For big urgent data, the owner node does not tend to deliver these directly using an interrupt data frame; instead, it sends a GTS request to the coordinator using an interrupt data frame. Next, the big urgent data will be transmitted using the GTS assigned by the coordinator. The last FCS field is used to carry the frame check sequence.

**Figure 3:**
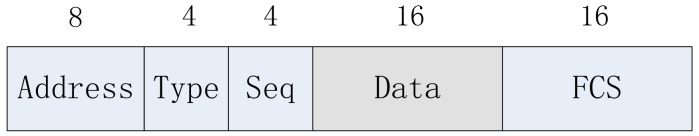
Structure of interrupt ack frame.

Figure 3 illustrates the structure of the frame transmitted in the ack section of an interrupt slot. We call such a frame an interrupt ack frame in this paper. The structure of an interrupt ack frame is similar to the structure of an interrupt data frame. The only difference lies in the length of the data field. The data carried by the interrupt ack frames are generally small-sized data, such as acknowledgments of data frames and network commands. The biggest data carried by the interrupt ack frame are the synchronization data encapsulated in a “SYNC” frame. Such data are used by the coordinator to denote how many time units have elapsed since the start of the current superframe, and these data are received by all nodes to synchronize their clocks with the coordinator.

### 3.3. Operations of I-MAC

This subsection introduces how the interrupt slots are used to deliver the urgent data. From above, there are two types of interrupt slots, upload interrupt slots and download interrupt slots. Correspondingly, the operations of I-MAC can be divided into two types, the download operations and upload operations, which are introduced respectively in the following. To carry out the operations, the coordinator and every node need to maintain a list that stores the urgent data and network commands. The data are sorted and stored in the list according to their priorities. The head of the list is the data with the highest priority, which will be transmitted when the coordinator or node has the chance to transmit its urgent data.

**Figure 4:**
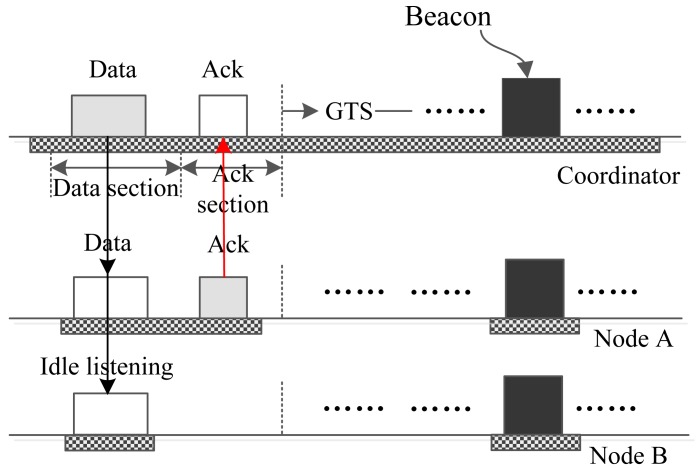
The coordinator sends small data to one node. GTS, guaranteed time slot.

Figure 4 reveals the process by which the coordinator sends one small urgent data frame to one node. In the data section, the coordinator encapsulates the data or command in a frame and sends out the frame, and all of the nodes wake up to receive the packet and check the address of the frame. If a node finds that the frame is not sent to it, then this node abandons the frame and goes to sleep, just as Node B does in the figure. If a node finds that it is the destination of the packet, it receives the packet and acknowledges it in the following ack section, just like Node A does in Figure 4.

**Figure 5:**
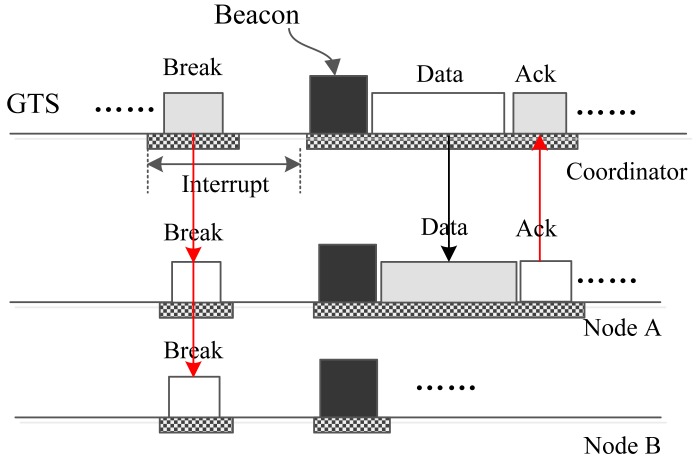
The coordinator sends big data to one node.

Suppose the coordinator has urgent big urgent data whose destination is Node A. In this case, the coordinator cannot send the data directly to the node using an interrupt slot due to the size of the data. To cope with this problem, the coordinator constructs a “BREAK” frame and broadcasts it in the data section of a download interrupt slot. The address of the frame is set to be the broadcast address. Upon receiving this frame, all nodes break the current superframe and begin a new superframe. In the new superframe, the coordinator assigns a download time slot to Node A using the beacon frame. Then, the coordinator sends the data to Node A, and node A replies with an acknowledge frame using the assigned time slot. The whole operation process is illustrated in Figure 5.

**Figure 6:**
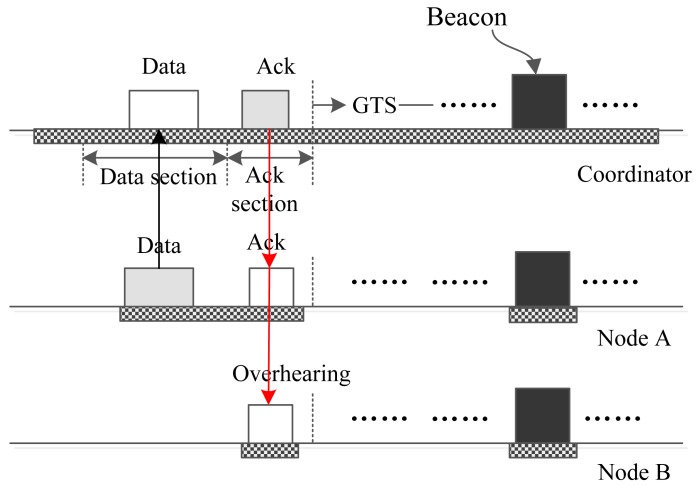
Only one node sends small data to the coordinator.

With regard to an upload interrupt slot, several cases may occur along with the data priority, data size and the number of data frames sent in the data section of the interrupt slot. We first examine the case that there is only one Node A who wants to send small data to the coordinator using an upload interrupt slot. On this occasion, the data can be transmitted completely by the data section of an upload interrupt slot. Therefore, Node A creates a frame and sends it to the coordinator in the data section during which the coordinator is in the receiving state. Upon accepting the frame, the coordinator acknowledges it in the following ack section. Node A accepts the ack packet and knows that the data packet has been accepted by the coordinator successfully. For another Node B, it keeps sleeping during the data section, because it does not have data to send. However, in the following ack section, it wakes up to receive the packet that may be sent by the coordinator. Once Node B finds that the packet is not sent to it, Node B abandons the frame and goes back to sleep. Figure 6 shows the whole process.

**Figure 7:**
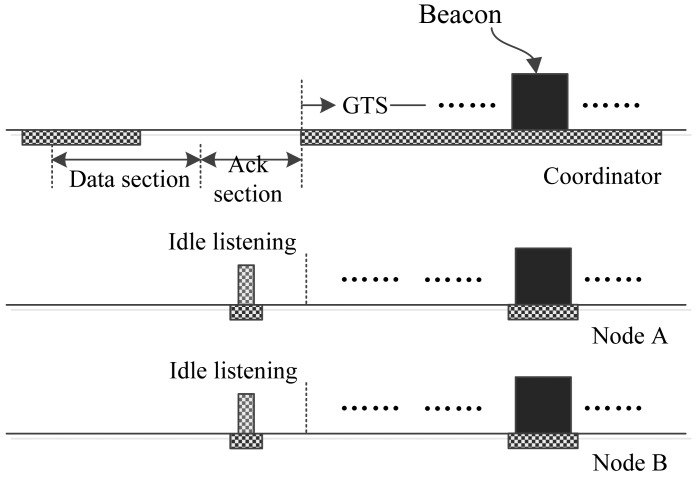
No node sends data to the coordinator.

It is possible that there is not a node with data to send during the data section of an upload interrupt slot. Figure 7 illustrates such a case. On this occasion, all of the nodes keep silent in the data section. The coordinator listens to the channel to receive a packet that may be sent by a node. Once the coordinator judges that there is not a packet for it, it stops listening and goes to sleep. If the coordinator does not have a “BREAK” or “SYNC” item on the head position of its lists, it keeps on sleeping in the rest of the interrupt slot. If the coordinator has such an item, then it may create a frame and send it in the ack section. It is assumed in Figure 7 that the coordinator does not have such an item. On this occasion, all of the nodes will listen to the channel for a while, then find that there is not a frame being transmitted, and then, the nodes will turn off their antennae to save energy.

**Figure 8:**
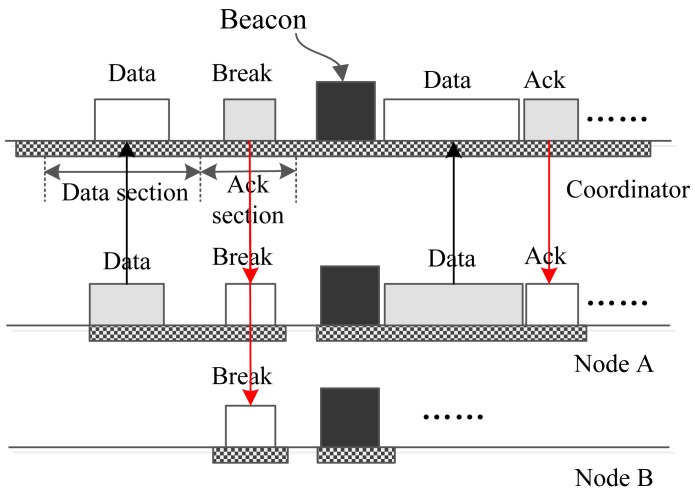
Only one node sends big data to the coordinator.

**Figure 9:**
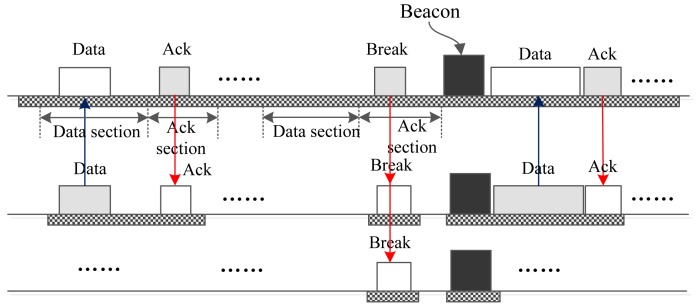
The case that a break is postponed.

Now, let us take a look at how I-MAC conveys a big urgent datum. As illustrated in Figure 8, if node A has big urgent data to transmit, it sends a GTS request packet to the coordinator in the data section of an upload interrupt section. The GTS request contains the size and priority *p*_1_ of the data. Upon accepting the GTS request, the coordinator examines the priority *p*_2_ of the data that will be transmitted in the following GTS. The coordinator compares *p*_1_ and *p*_2_. If *p*_1_ is smaller than or equal to *p*_2_, then the coordinator stores an item in the data list and replies to Node A with an ack packet in the following ack section. The coordinator may break the current superframe with a “BREAK” command in some later interrupt slot, just as Figure 9 depicts. If *p*_1_ is bigger than *p*_2_, then the coordinator constructs a “BREAK” packet and broadcasts this packet in the following ack section. Upon accepting this packet, all nodes start a new superframe. In the new superframe, a GTS at the beginning is assigned to Node A so that it can transmit its data with the highest priority first.

Figure 9 shows a case of a postponed break of the superframe. As shown by the figure, after accepting the frame in the data section, the coordinator does not broadcast a “BREAK” frame in the ack section; instead it replies to Node A with an ack frame. The “BREAK” frame is postponed to a later download or upload interrupt slot. Figure 9 gives the case that the “BREAK” frame is postponed to an upload interrupt slot. After this, the superframe is broken, and another new superframe is started; then, Node A sends the data to the coordinator in the first GTS of the new superframe. The coordinator also can use a download interrupt slot to broadcast the “BREAK” frame. The process is similar to Figure 9 and is presented in this paper.

**Figure 10:**
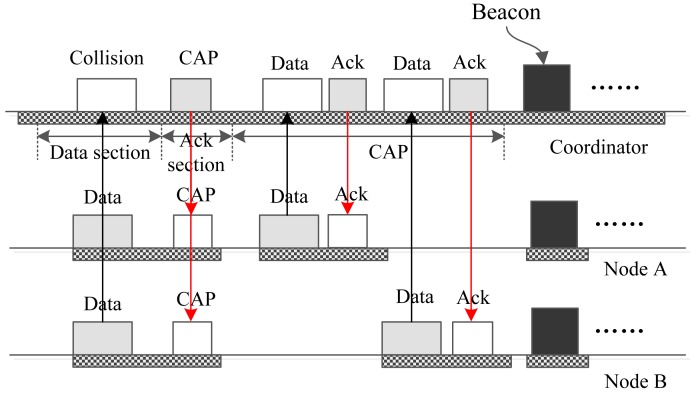
The case that there is a collision.

It is worth noting that there may be two or more nodes that have data to send in the data section of an upload interrupt slot. In this case, the frames will collide with each other. Figure 10 gives a case in which there are two nodes, A and B, sending their data to the coordinator in the same data section. As a result, the coordinator cannot receive the packets successfully. The coordinator distinguishes the reason for the communication failure. If it recognizes that the failure was caused by the collision of packets sent by multiple nodes, it broadcasts a “CAP” packet in the following ack section. A field indicating the length of the coming CAP period is included in the packet. Upon receiving the “CAP” packet, all of the nodes and the coordinator enter a CAP period in which the nodes compete with each other using CSMA/CA to send the data to the coordinator. After the CAP period, the coordinator broadcasts a new beacon frame, and all of the nodes receive the beacon. Then, a new superframe begins, and the network continues running.

The coordinator can use the ack section of an upload interrupt slot or the data section of a download interrupt slot to broadcast a “SYNC” frame to all nodes. This is important for I-MAC. Because I-MAC uses a long superframe structure to save energy, this may introduce long clock drift. As a consequence, the clocks of the coordinator and nodes may be unsynchronized if no synchronization measures are taken. This may cause the waste of energy and even the failure of communication. Therefore, I-MAC needs to use interrupt slots to transmit “SYNC” frames to synchronize the clocks of the coordinator and nodes. This operation is similar to the operations described above and is not discussed in this paper.

## 4. Performance Analysis

This section analyzes the energy efficiency, delivery latency and time slot utilization efficiency in transmitting the urgent data for IEEE 802.15.4 and I-MAC. The time slot utilization efficiency measures how much time is used to deliver the urgent data. For both protocols, it is assumed that the periodic data are conveyed using the GTS allocated by the same algorithm. Consequently, the two protocols have the same energy efficiency, delivery latency and time slot usage efficiency for transmitting periodic data. Therefore, we only consider the energy efficiency, delivery latency and time slot usage efficiency in delivering the urgent data. For 802.15.4, the urgent data are delivered by the CAP period. For I-MAC, the urgent data are conveyed by the interrupt time slots or the CAP period. Therefore, although the periodic data are delivered by GTS, the GTS request and release commands are delivered by the CAP period or the interrupt slots, because they belong to the urgent data. Both the upload interrupt slots and download interrupt slots are used to convey the urgent data. Yet, the major contributor of the urgent traffic is the nodes, because the urgent data generated by the nodes outdistance the coordinator. Therefore, we only consider the upload interrupt slots in the analysis and simulation.

The wireless body area network can be represented as *WBAN* = {*C, N*_1_, *N*_2_, …, *N*_n_}, in which *C* is the coordinator and {*N*_1_, *N*_2_, …, *N*_n_} is the set of the nodes. It is assumed the urgent data are generated according to the Poisson process. *E_B_* = {λ_1_*_B_*, λ_2_*_B_*, …, λ*_nB_*} is the set of the average big urgent data arrival rates of all nodes. *E_S_* = {λ_1_*_S_*, λ_2*S*_, …, λ*_nS_*} is the set of the average small urgent data arrival rates of all nodes. We assume that all the processes are independent. According to the theory of random processes, the big and small urgent data processes of a node can form a new Poisson process. Therefore, the urgent data arrival rates of all nodes can be expressed as *E* = {λ_1_, λ_2_, …, λ_*n*_}, where λ*_i_* = λ_i_*_B_* + λ*_iS_*. The big urgent data arrival processes of all nodes can form a new process with arrival rate $\lambda_{B}=\overset{n}{\underset{i=1}{\text{∑}}}\lambda_{iB}$. The small urgent data arrival processes of all nodes can form a new process with arrival rate $\lambda_{S}=\overset{n}{\underset{i=1}{\text{∑}}}\lambda_{iS}$. All of the urgent data arrival processes can form a new process with arrival rate λ = λ*_B_* + λ*_S_*.

### 4.1.802.15.4 Performance Analysis

The analytical approach used in [30,31] is adopted in this paper. By dividing the consumed energy into time periods, e.g., the beacon interval, the energy efficiency of different protocols can be expressed as energy power. This provides an effective way to compare the energy efficiency. Using this analytical approach, we first analyze the performance of 802.15.4 in the following.

Under beacon mode, the nodes need to wake up periodically to receive the beacon frame. The time-base tolerance of the transmitter and receiver must be taken into account [31]. Therefore, the average beacon duty cycle of a receiver is determined by:

(1) $$DC=\frac{2\left({ɛ}_{\text{TX}}+{ɛ}_{\text{RX}}\right)BI+T_{\text{Beacon}}+T_{TX\_wu}+T_{RX\_wu}}{BI}$$

where *BI* stands for the beacon interval, 2 (*ε*_TX_ + *ε_RX_*) *BI* is the clock drift of the transmitter and receiver, *T_Beacon_* is the transmission time of a beacon frame and *T_TX_wu_* and *T_RX_wu_* are the warm-up times of the transmitter and receiver. *DC* indicates the ratio of the time for receiving the beacon frame to the beacon interval.

Using 802.15.4, a node transmits its urgent data to the coordinator using the CAMA/CA scheme during the CAP period. The receiving processes related to sending a datum to the coordinator include receiving the acknowledge frame, the clear channel assessment (CCA) operation and the receiver warming up process. The average receiver power for sending an urgent datum from a node to the coordinator is presented by:

(2) $$P_{RX\_AV}=\left(DC+\frac{T_{\text{ack}}+\left(2+R\right)\cdot T_{RX\_wu}+2R\cdot T_{\text{CCA}}}{T_{\text{Event}}}\right)P_{RX}$$

where *T_Ack_* is the acknowledgment frame duration, *T_RX_wu_* represents the receiver warm-up time, *T_CCA_* is the clear channel assessment time, *R* denotes the average number of back-offs [26], *P_RX_* is the receiver power in receiving mode and *T_Event_* is the average interval that all of the nodes generate urgent data, which is equal to nλ.

Small urgent data are directly transmitted to the coordinator during the CAP period or interrupt slots. Yet, for big urgent data, the node first sends a GTS request to the coordinator, and then, the data are conveyed using the assigned GTS. For the sake of convenience, we do not consider the energy used to transmit the whole of the big data; instead we only take the energy for transmitting the GTS requests into account. Based on this, the average power for transmitting a data frame is given by:

(3) $$P_{TX\_AV}=\left(\frac{T_{\text{Data}}+T_{TX\_wu}}{T_{\text{Event}}}\right)P_{TX}$$

where *T_Data_* represents the time duration of a data frame, *T_TX_wu_* is transmitter warm-up time and *P_RX_* denotes the transmitter power in transmitting mode.

In this paper, the data delivery delay is defined as the time elapsed from the generating time of the data to the arrival time of these data or the corresponding GTS request to the coordinator. Using 802.15.4, all of the urgent data are delivered during the CAP period. We assume that the urgent data generated during the CAP period can be delivered immediately, and the data generated during the CFP period will be delivered in the next CAP period. Based on this, the delivery delay of a data generated during the CAP period is:

(4) $$T_{1}=T_{\text{CSMA}}+T_{\text{Data}}$$

where *T_Data_* is the transmission time of a data frame and *T_CSMA_* is the average medium access time, which is given by the following Equation [30]:

(5) $$T_{\text{CSMA}}=T_{TX\_wu}+R\cdot T_{\text{CCA}}+f\left(R,T_{BO\left(\alpha \right)}\right)$$

The average delivery delay of the data generated out of the CAP period is given by:

(6) $$T_{2}=\frac{BI-T_{\text{CAP}}}{2}+T_{\text{CSMA}}+T_{\text{Data}}$$

Based on the above, the average delivery delay of the urgent data can be expressed as:

(7) $$D_{802.15.4}=P_{1}\cdot T_{1}+P_{2}\cdot T_{2}=P_{1}\cdot \left(T_{\text{CSMA}}+T_{\text{Data}}\right)+P_{2}\cdot \left(\frac{BI-T_{\text{CAP}}}{2}+T_{\text{CSMA}}+T_{\text{Data}}\right)$$

where *P*_1_ and *P*_2_ are the probabilities that urgent data are generated during the CAP period or out of the CAP period. *P*_1_ and *P*_2_ can be expressed as the following two equations:

(8) $$P_{1}=\frac{T_{\text{CAP}}}{BI}$$

(9) $$P_{2}=1-P_{1}=1-\frac{T_{\text{CAP}}}{BI}$$

The time slot utilization efficiency is measured by the ratio of the time spent on delivering the urgent data to the whole time. For 802.15.4, the time related to conveying the urgent data is the beacon time and the CAP period. From the IEEE 802.15.4 standard, the CAP period at least occupies 1/16 of the active period of the superframe, and we assume that this is enough for transmitting the urgent data. Let the duty cycle of the active period be *DC_Active_*; then, the time slot usage of 802.15.4 can be expressed as:

(10) U802.15.4=DC+116·DCActive

### 4.2. I-MAC Performance Analysis

In a WBAN that adopts the I-MAC protocol, the coordinator and the nodes transmit the urgent data using the operations described in Section 3. We first calculate the power spent by a node for receiving operations. A node may receive regular beacons or beacons that are broadcast after superframes are broken. During the ack section of every interrupt slot, every node needs to wake up to receive the packet that may be broadcast by the coordinator, no matter whether the node has just transmitted data or not. If a node finds that there is not a transmitting packet or a packet is not sent to it, it abandons the receiving operation. We assume this operation spends Ti_down2 time to complete. If there is only one small urgent datum generated during the interrupt interval, the node that generated the datum needs to receive the acknowledgment frame. If there is one big urgent datum or more than one datum generated, then the current superframe will be broken, and a new beacon will be broadcast. On this occasion, all nodes need to receive the new beacon. Therefore, the average power consumed by a node for receiving can be expressed as the following equation:

(11) PNode_RX_AV=(DC+Ti_ack+TRX_wuTEvent+Ti_ack2+TRX_wu+Px=1,S·Ti_ack/2n+(Px=1,B+Px≥2)(Ti_ack2+TBeacon+TRX_wu)IInt)PRX

where *DC* denotes the duty cycle of the regular beacon, $\frac{T_{i\_\text{ack}}+T_{RX\_wu}}{T_{\text{Event}}}$ represents the duty cycle contributed by receiving the acknowledgment frame for every urgent data and *P*_*x*=1,*S*_, *P*_*x*=1,*B*_ and *P*_*x*≥2_ are the probabilities that only one small, only one big and more than one urgent datum are generated during the interrupt interval. *P*_x=1,*S*_, *P*_*x*=1,*B*_ and *P*_*x*≥2_ are represented by the following three equations:

(12) $$P_{x=1,B}=\frac{\lambda_{B}\cdot I_{\text{Int}}\cdot e^{-\left(\lambda_{B}+\lambda_{S}\right)\cdot I_{\text{Int}}}}{\lambda_{B}+\lambda_{S}}=\frac{\lambda_{B}\cdot I_{\text{Int}}\cdot e^{-\lambda \cdot I_{\text{Int}}}}{\lambda }$$

(13) $$P_{x=1,S}=\frac{\lambda_{S}\cdot I_{\text{Int}}\cdot e^{-\left(\lambda_{B}+\lambda_{S}\right)\cdot I_{\text{Int}}}}{\lambda_{B}+\lambda_{S}}=\frac{\lambda_{S}\cdot I_{\text{Int}}\cdot e^{-\lambda \cdot I_{\text{Int}}}}{\lambda }$$

(14) $$P_{x\geq 2}=1-P_{x=0}-P_{x-1}=1-e^{-\lambda \cdot I_{\text{Int}}}-\lambda \cdot I_{\text{Int}}\cdot e^{-\lambda \cdot I_{\text{Int}}}$$

As for the power spent on transmitting the urgent data to the coordinator, the data frames need to be transmitted twice if there is more than one urgent datum generated during an interrupt interval. Therefore, the average power consumed by a node for transmitting can be expressed as the following equation:

(15) $$P_{TX\_AV}=\left(\frac{P_{x=1}\cdot \left(T_{\text{Data}}+T_{TX\_wu}\right)+\overset{n}{\underset{k=2}{\text{∑}}}P_{x=k}\cdot 2k\left(T_{\text{Data}}+T_{TX\_wu}\right)}{n\cdot I_{\text{Int}}}\right)P_{TX}$$

where *P*_*x*=*k*_ is the probability that there are kurgent data generated during the interrupt interval. *P*_*x*=*k*_ is given by the following equation:

(16) $$P_{x=k}=\frac{{\left(\lambda \cdot I_{\text{Int}}\right)}^{k}}{k!}\cdot e^{-\lambda \cdot I_{\text{Int}}}$$

Because I-MAC uses interrupt slots to deliver the urgent data, the data delivery delay of I-MAC strongly depends on the interrupt interval. The average time urgent data has to wait before they can be transmitted is IInt2. The time for a node to transmit urgent data to the coordinator is *T_Data_*. If there is more than one datum generated during an interrupt interval, extra delay is introduced by an extra CAP period. The average delivery delay of I-MAC is given by:

(17) $$D_{I-\text{MAC}}=\frac{I_{\text{Int}}}{2}+T_{\text{Data}}+T_{i\_\text{ack}}+\frac{P_{x\geq 2}\cdot I_{\text{CAP}}}{2\left(P_{x\geq 2}+P_{x=1}\right)}$$

The lengths of *I_Int_* and *I_CAP_* are determined during the working process of I-MAC. The coordinator counts the number of urgent data and performs a statistical treatment. From that, the coordinator can obtain the data arrival rates of all nodes. Then, the coordinator can calculate an appropriate interrupt slot interval. Based on our experiments, an interrupt slot interval with length *I_Int_* is appropriate if λ· *I_Int_* < 1. When a collision occurs, the coordinator estimates the number of frames that causes the collision by λ and *I_Int_*. Then, it chooses a longer length for the CAP period, so that it is long enough for transmitting the data.

As for the data of high priority, that is the data with a high real-time requirement, these data are always placed at the head part of the data list and prioritized to be transmitted first. Therefore, the delay of the data of high priority is less than *D_I−MAC_*.

The time slot usage of I-MAC involves the beacon periods, the interrupt slots and the CAP periods. Therefore, it can be calculated by the following equation:

(18) $$U_{I-\text{MAC}}=DC+\frac{T_{i}}{I_{\text{Int}}}+\frac{P_{x\geq 2}\cdot I_{\text{CAP}}}{\left(P_{x\geq 2}+P_{x=1}\right)I_{\text{Int}}}$$

## 5. Performance Evaluation

The purpose of I-MAC is to reduce the energy consumption, increase the use ratio of time slots and guarantee the real-time demand of urgent event packets in the meantime. Around these three aspects, the energy efficiency, data delivery delay and time slot usage efficiency of 802.15.4 and I-MAC are evaluated and compared in this section. Both the numerical analysis in Section 4 and the simulation results are used for the evaluation and comparison. OMNeT++ [32] is adopted as our simulation tool. We first fix the urgent data occurrence interval, change the beacon interval and interrupt slot interval, compare the energy efficiency, data delivery delay and time slot usage efficiency of both protocols and examine how these performances change with the intervals. Then, the beacon interval and interrupt slot interval are fixed, and the data occurrence interval is changed. Then, we compare the performances of both protocols and examine how the performances change with the data occurrence interval.

A star WBAN composed of one coordinator and 20 nodes is used in the simulation. The hardware parameters are specified according to CC2420 [33] and IEEE 802.15.4. We assume the beacon lengths of 802.15.4 and I-MAC are 30 bytes and 34 bytes, because I-MAC has a more complex beacon structure than 802.15.4. To transmit two beacons, 0.96 ms and 1.088 ms are needed, respectively. The acknowledgment frame length of 802.15.4 is five bytes, which needs 0.15 ms to be transmitted. For I-MAC, the data frame and acknowledgment frame used for the urgent data are 10 bytes and six bytes, which need 0.32 ms and 0.192 ms to be transmitted. The data section and ack section of an interrupt slot are set to be 0.384 ms and 0.256 ms, which are enough for delivering the data frame and acknowledgment frame. The parameters are summarized in Table 3.

From the above description, the energy efficiency, data delivery delay and time slot utilization efficiency strongly depend on the beacon interval of 802.15.4 and the interrupt interval of I-MAC. We first examine how these performances change with the intervals. To observe the effect of the change of the intervals clearly, the average urgent data occurrence interval is fixed to be 20 min, a quite high value that does not affect the observation of the effect of the change of the intervals. For the sake of convenience, all nodes are set to use the same average urgent data occurrence interval.

Two MAC schemes are compared in this section. The first scheme is 802.15.4, and the other one is I-MAC. For I-MAC, three variants take the same interrupt interval and different beacon intervals. Let BI be the beacon interval and *I_Int_* be the interrupt slot interval, then the number of interrupt slots in a superframe can be calculated by NI=BIIInt. From this, the three I-MAC schemes compared are differentiated by *NI*.

**Figure 11:**
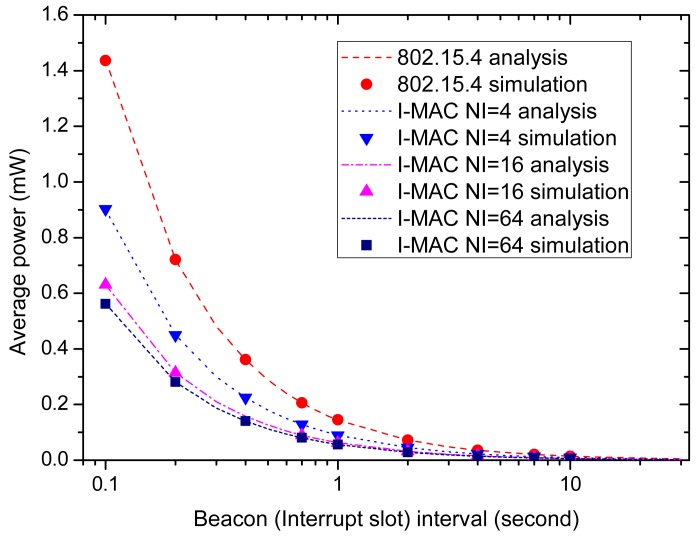
The effects of the beacon interval and interrupt interval on node average power.

Figure 11 shows how the average power of a node used under different MAC schemes changes with the beacon interval and the interrupt interval. Because the average data occurrence interval set in the simulation is quite long, the energy consumed for transmitting the urgent data only contributes little to the whole consumed energy. The main contributors of the energy consumption include the reception of the beacon frames and the idle listening during the ack section of the interrupt slots. From the figure, the average powers of all of the MAC schemes decrease as the beacon interval and interrupt interval increase. The reason for this is the numbers of beacons and interrupt slots decrease as the beacon interval and interrupt interval increase. It also can be observed that all I-MAC variations have higher energy efficiency than 802.15.4, and the average power of an I-MAC variation with a longer beacon interval is lower than an I-MAC variation with a longer beacon interval. All of these can be explained by the reduction of beacons sent in these schemes.

**Figure 12:**
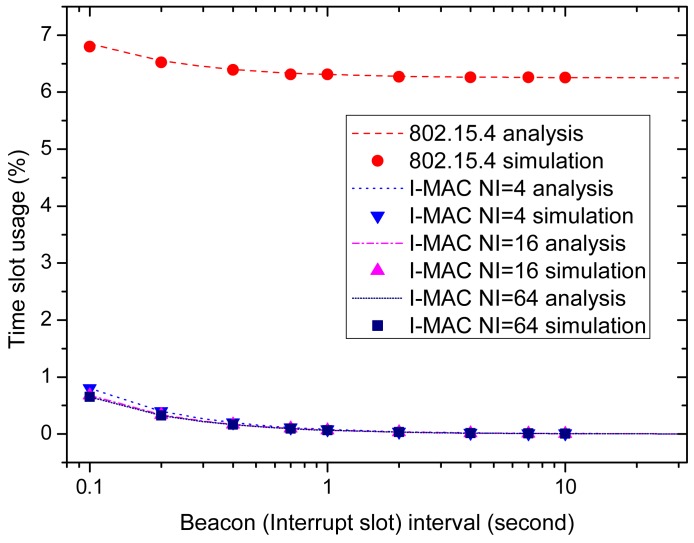
The effects of the beacon interval and interrupt interval on time slot usage.

Figure 12 shows the time slot utilization efficiencies of all of the MAC schemes. This performance indicator indicates how much time resource is used to transmit the urgent data for every scheme. For 802.15.4, the beacons and CAP periods are the factors that affect this indicator; while for I-MAC, the beacons and the interrupt slots are the factors. It can be seen from the figure that as the beacon interval and interrupt interval increase, the time slot usages of all of the MAC schemes decrease with the reduction of beacons. For all MAC schemes, the duty cycle of the coordinator is 100% due to the existence of monitoring applications of high data rates. For 802.15.4, to deliver the urgent data, a CAP period that at least occupies one of the total 16 time slots needs to be configured in a superframe. In this paper, such a CAP period is enough for transmitting the urgent data. Therefore, when the beacon interval increases, the time slot usage tends to 1/16. Compared to 802.15.4, the short interrupt slots exploited by I-MAC only occupy a small fraction of the total time slots. Therefore, I-MAC has higher time slot utilization efficiency than 802.15.4. As for the three I-MAC variations, the scheme with more interrupt slots has higher time slot utilization efficiency, but the advantage is not obvious.

**Figure 13:**
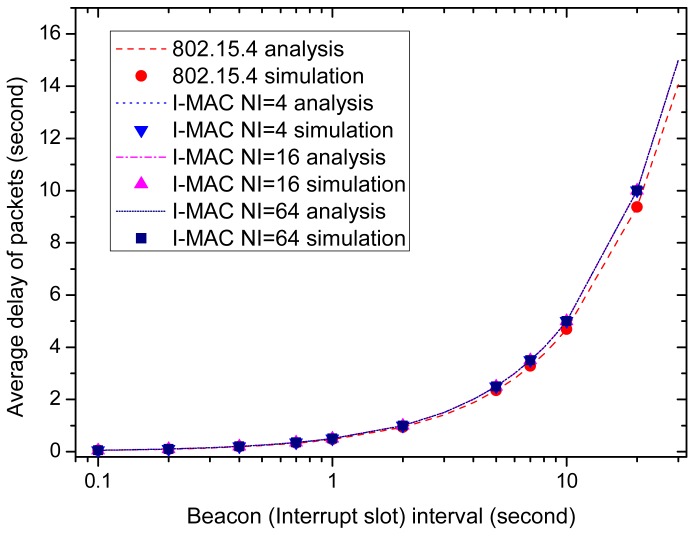
The effects of the beacon interval and interrupt interval on average packet delay.

It is revealed by Figures 11 and 12 that a longer beacon interval and interrupt interval can bring higher energy efficiency and time slot utilization efficiency. However, along with these advantages, there also comes a disadvantage, that is long data delivery delay. Figure 13 shows how the average packet delay changes with the change of the beacon interval and interrupt interval. It can be observed that the data delivery delay increases as the beacon interval and interrupt interval increase. This is because the data have to wait longer as the intervals increase. The delay of 802.15.4 is a little lower than BI2, and the delays of all I-MAC variations are approximately IInt2. The delivery delay of I-MAC is a little longer than 802.15.4, especially when the beacon interval and interrupt interval have large values.

From Figure 13, it can be seen that the beacon interval and interrupt interval should be set with an appropriate value in order to meet the real-time requirements. Generally speaking, if the average delay is required to be smaller than a value *D*, then the beacon interval and interrupt interval can be set to be a value smaller than 2*D*. If the delay is required to be strictly smaller than *D*, then the beacon interval and interrupt interval can be set to be a value smaller than *D*.

After examining the effects of the beacon interval and interrupt interval on the performances, we fix the beacon interval and interrupt interval and examine the effect of the data generation interval on the performances of all of the MAC schemes. We assume the average urgent data delivery delay is 0.3 s, and the beacon intervals and interrupt intervals of all of the MAC schemes are all set to be 0.5 s, which is sufficient to meet the delay requirement according to Figure 13. Like the above simulations, all nodes are set to use the same average urgent data occurrence interval for the sake of convenience. Ten percent of the urgent data is big data that may break the running superframe and start a new one. The average data occurrence interval varies from 1 s to 10,000 s, and this means that the number of urgent data generated in one interrupt interval varies from 10 to 0.001. The effects of the average data occurrence interval on the average power consumed by a node for delivering the urgent data, the time slot utilization efficiency and the data delivery delay are examined and the performances of all of the MAC schemes are compared in the following part.

**Figure 14:**
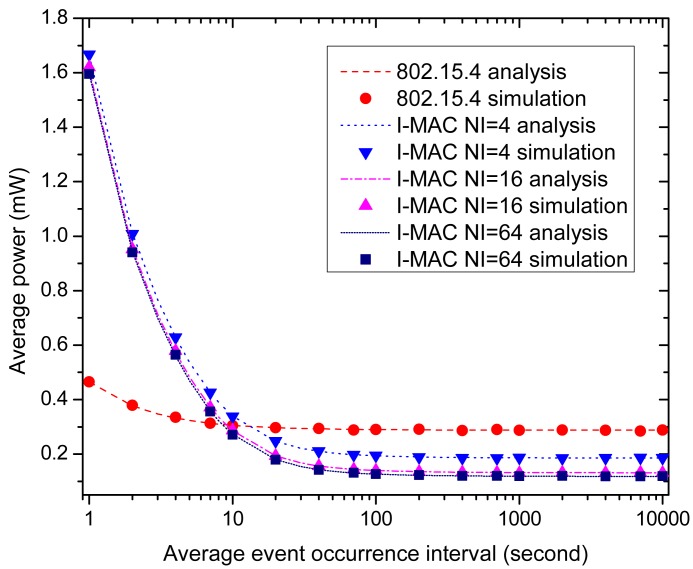
The effect of the average event occurrence interval on the average power.

Figure 14 displays the effect of the average data occurrence interval on the average power consumed by a node. As the figure shows, three I-MAC variations cost high energy consumption when the data occurrence interval is small, and the energy consumptions of all three variations decrease rapidly as the data occurrence interval grows. In contrast, the energy consumption of 802.15.4 is only a little high when the data occurrence interval is small, and the average power decreases slowly as the data occurrence interval grows. The average powers of three I-MAC variations are far higher than 802.15.4, as the data occurrence interval takes small values; yet, as this interval grows, the average powers of three I-MAC variations quickly become smaller than 802.15.4. The reason to explain the above phenomena lies in the frequent collisions of the urgent data frames and so incurred CAP periods and breaks of the superframe. These operations cause extra retransmissions of the urgent data frames and extra beacon frames, which then incur high energy consumption. From Figure 14, it can be observed that 10 s is a critical point. At this point, there is averagely one urgent datum generated in 0.5 s, *i.e.*, the interrupt interval.

**Figure 15:**
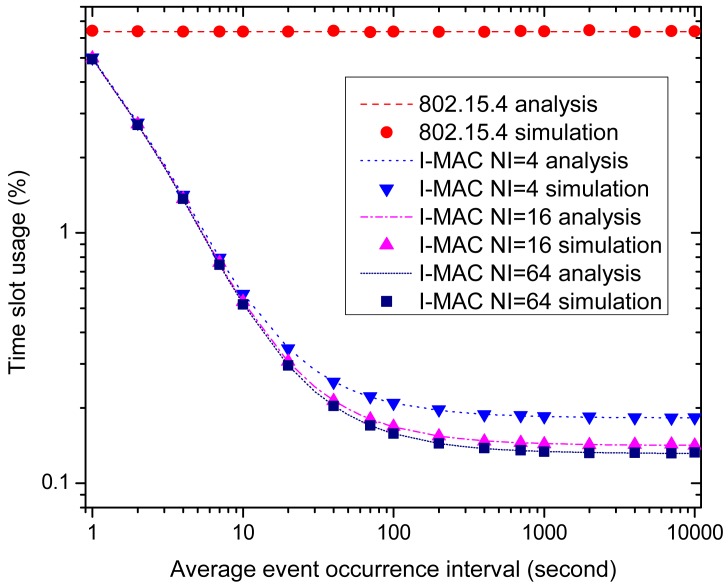
The effect of the average event occurrence interval on time slot usage.

The time slot utilization efficiencies of all of the MAC schemes are illustrated by Figure 15. For 802.15.4, the beacons and CAP periods remain the same as the average event occurrence interval changes; therefore, the time slot usage of 802.15.4 remains constant and is unacted on the change of the occurrence of the urgent events; while for three I-MAC variations, the time slot usages exhibit a rapid decrease as the average event occurrence interval changes from small to big, just like what is exhibited by the average powers of three I-MAC variations in Figure 14. This can also be explained by the fact that a small average urgent event interval gives rise to frequent collisions of the urgent data frames, and the CAP periods and beacon frames so caused will increase the time usage for transmitting the urgent data and extra beacon frames.

**Figure 16:**
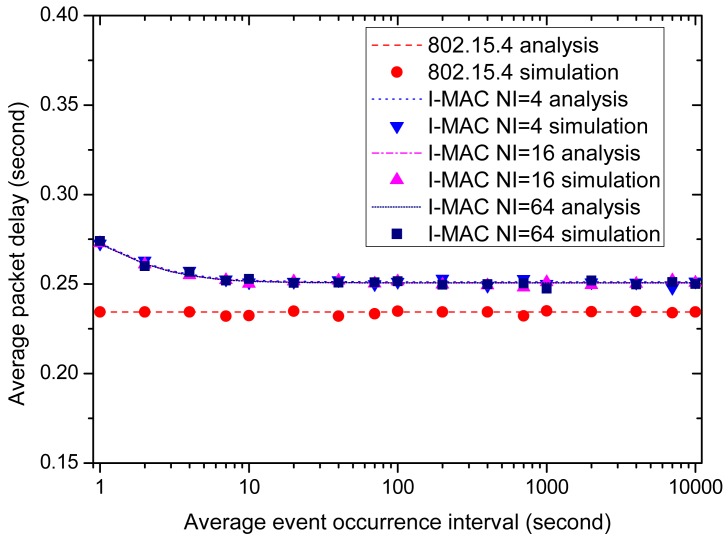
The effect of the average event occurrence interval on average packet delay.

Finally, let us examine the effect of the average event occurrence interval on the average packet delay. Figure 16 shows and compares the average packet delays of all of the MAC schemes. As the figure displays, all of the MAC schemes can satisfy the time delay requirement of the urgent data, which is 0.3 s. For 802.15.4, the average packet delay remains constant at about 0.235 s as the average event occurrence interval varies 1 s to 10,000 s. As for I-MAC, the average packet delays of the three variations exhibit a rise when the average event occurrence interval takes small values and decrease to a little above 0.25 s as the average event occurrence interval grows big. These rises can also be explained by the extra CAP periods and beacon frames incurred by the collisions of the urgent data frames.

To sum up, when the urgent event occurrence frequency is not high, I-MAC can effectively improve the energy efficiency and time usage efficiency in transmitting the urgent data, as well as satisfy the time delay requirement at the same time. However, when the urgent event occurrence frequency becomes high, the energy efficiency and time usage efficiency of I-MAC exhibit severe performance degradation, because of frequent collisions of the urgent data frames. Therefore, I-MAC is not effective when the urgent event occurrence frequency is high. According to our experiments, the average event occurrence interval at which there is an average of one datum that can be generated is the critical point. If the interval is beyond this point, I-MAC can be adopted as an effective way to improve the network performance.

## 6. Conclusions

Targeting the medical monitoring applications of WBANs, I-MAC is proposed to improve the energy efficiency and time slot utilization efficiency, as well as to meet the data delivery delay requirement at the same time. I-MAC adopts a long superframe structure and uses short interrupt slots to deliver the urgent data. To some extent, the interrupt slots of I-MAC can be viewed as a substitute of the beacon and CAP period. The energy efficiency of I-MAC is obtained by the reduction of beacon frames. The time slot utilization efficiency of I-MAC is obtained by substituting the CAP periods with the interrupt slots. Experiments show that I-MAC works effectively when the urgent event occurrence frequency is not high. According to the experiments, the average event occurrence interval at which there is an average of one datum that can be generated is the critical point. If the interval is beyond this point, I-MAC can be adopted as an effective way to improve the network performance.

## Figures and Tables

**Table 1:** Data rates and delay demands of WBAN applications.

Application	Data Rate	Delay
ECG (12 leads)	288 kbps	250 ms
ECG (6 leads)	71 kbps	250 ms
EMG	320 kbps	250 ms
EEG (12 leads)	43.2 kbps	250 ms
Blood saturation	16 bps	250 ms
Temperature	120 bps	250 ms
Glucose monitoring	1600 bps	250 ms
Motion sensor	35 kbps	250 ms
Cochlear implant	100 kbps	250 ms
Artificial retina	50–700 kbps	250 ms
Audio	1 Mbps	100 ms
Video	<10 Mbps	100 ms
Voice	50–100 kbps	100 ms

**Table 2:** Comparison between TDMA and CSMA/CA.

Comparison Item	TDMA	CSMA/CA
Power consumption	Low	High
Bandwidth utilization	Maximum	Low
Preferred traffic level	High	Low
Dynamic (network change)	Poor	Good
Effect of packet failure	Latency	Low
Synchronization	Essential	–

**Table 3:** Parameters used in the simulation. CCA, clear channel assessment.

Parameter	Value
Voltage supply	1.8 V
Receiver current	20 mA
Transmitter current (0 dBm)	17.4 mA
Receiver\transmitter start up time	1.4 ms
Clock drift rate	30 ppm
CCA time	0.125 ms
Bit rate	250 kbps
802.15.4 beacon	0.96 ms
I-MAC beacon	1.088 ms
802.15.4 ack	0.16 ms
Interrupt data section	0.384 ms
Interrupt ack section	0.256 ms

## Supplementary Materials

Supplementary materials can be accessed at: http://www.mdpi.com/1424-8220/15/6/12906/s1.
